# Dendritic overgrowth and elevated ERK signaling during neonatal development in a mouse model of autism

**DOI:** 10.1371/journal.pone.0179409

**Published:** 2017-06-13

**Authors:** Ning Cheng, Fawaz Alshammari, Elizabeth Hughes, Maryam Khanbabaei, Jong M. Rho

**Affiliations:** 1 Developmental Neurosciences Research Program, Alberta Children’s Hospital Research Institute (ACHRI), Cumming School of Medicine, University of Calgary, Calgary, Alberta, Canada; 2 O’Brien Centre for the Bachelor of Health Sciences, Cumming School of Medicine, University of Calgary, Calgary, Alberta, Canada; 3 Departments of Pediatrics, Clinical Neurosciences, Physiology & Pharmacology, Alberta Children’s Hospital Research Institute (ACHRI), Cumming School of Medicine, University of Calgary, Calgary, Alberta, Canada; Centre National de la Recherche Scientifique, FRANCE

## Abstract

Autism spectrum disorder (hereafter referred to as “ASD”) is a heterogeneous neurodevelopmental condition characterized by impaired social communication and interactions, and restricted, repetitive activities or interests. Alterations in network connectivity and memory function are frequently observed in autism patients, often involving the hippocampus. However, specific changes during early brain development leading to disrupted functioning remain largely unclear. Here, we investigated the development of dendritic arbor of hippocampal CA1 pyramidal neurons in the BTBR T+tf/J (BTBR) mouse model of autism. BTBR mice display the defining behavioural features of autism, and also exhibit impaired learning and memory. We found that compared to control C57BL/6J (B6) animals, the lengths of both apical and basal dendrites were significantly greater in neonatal BTBR animals. Further, basal dendrites in the BTBR mice had higher branching complexity. In contrast, cross-sectional area of the soma was unchanged. In addition, we observed a similar density of CA1 pyramidal neurons and thickness of the neuronal layer between the two strains. Thus, there was a specific, compartmentalized overgrowth of dendrites during early development in the BTBR animals. Biochemical analysis further showed that the extracellular signal-regulated kinases (ERK) pathway was up-regulated in the hippocampus of neonatal BTBR animals. Since dendritic structure is critical for information integration and relay, our data suggest that altered development of dendrites could potentially contribute to impaired hippocampal function and behavior observed in the BTBR model, and that this might be related to increased activation of the ERK pathway.

## Introduction

Autism spectrum disorder (ASD) is an increasingly prevalent neurodevelopmental disorder, characterized by deficits in socio-emotional functions and language development, as well as repetitive and/or restrictive behaviours [1–4]. In addition, ASD has broad and heterogeneous clinical manifestations, which has been associated with many potential etiological factors including both genetic and environmental ones, making it challenging to investigate its neurobiological basis and find interventions for affected individuals. Currently, only co-morbid manifestations of the disorder can be alleviated, but not the core symptoms.

Efforts to identify consistent neural features of ASD have revealed larger brain volumes compared to age-matched controls, particularly in early childhood [5–11]. In addition, large amount of data point to age-dependent changes in structural and functional connectivity [12, 13], which may underlie the heterogeneous symptomatology seen in ASD. Together, these findings suggest that alterations in early neurodevelopment may contribute to disease pathogenesis, and that these early changes may be very dynamic.

Outgrowth of neuronal processes, including both dendrites and axons, is an important step during the critical window of early neurodevelopment. The elaborate structure of the dendritic arbor is a hallmark of neurons, and is a major defining factor of how input information is integrated and how synaptic plasticity is induced by the neuron [14, 15]. Not surprisingly, changes in dendritic structure occur in multiple psychiatric disorders, including ASD [16–18]. Collectively, the evidence to date, mostly from animal models of ASD, suggests that dendritic structure could be altered in ASD brain [19–26]. However, developmental trajectory and underlying mechanisms of these changes remain largely unclear. Nevertheless, previous studies on dendritic development have provided many molecular candidates, including neurotrophic factors [27–30], the cascade of mammalian target of rapamycin (mTOR) [31], and the pathway of extracellular signal-regulated kinases (ERK) [22, 25, 32, 33]. Notably, recent studies have shown that pharmacological inhibition of ERK signaling reverses increased dendritic arborization observed in two mouse models of ASD [22, 25].

Studies on neuronal density in brains of autistic individuals have yielded inconsistent results. While some reported elevated neuronal density in cortical areas and hippocampus of autistic individuals compared with typically developing controls [10, 34, 35], others observed similar densities between the two groups [35–37]. This may be due to small sample sizes, or heterogeneity of alterations in neuronal density in different brain regions or distinct cytoarchitectural domains. Interestingly, a recent study showed that overproduction of upper-layer neurons in the neocortex produced autism-like features in mice [38], emphasizing the potential importance of neuronal density in ASD.

The hippocampus is a well-studied structure in the vertebrate brain, due to its defined location, structure, and importance to learning and memory. Thus far, neuroimaging studies have not consistently reported difference in the overall volume of hippocampus between ASD and control groups [35, 39]. However, other studies have indicated that declarative memory in ASD patients is characterized by selective impairment in both encoding and retrieving of episodic memory, which is relational and contextual. In contrast, semantic memory, which is factual and item-based, is relatively preserved [39]. Some individuals with ASD even exhibit superior memory that depends on repetition and familiarity, but have difficulties with memory that uses relation and context. It has been hypothesized that these impairments may be due to alterations in hippocampal function and/or connectivity [39]. For example, recent studies reported that male carriers of the FMR1 premutation, who are at increased risk of developing ASD, showed altered hippocampal-prefrontal connectivity during memory encoding [40] and reduced hippocampal activation during memory recall [41]. These results suggest that more detailed anatomical and functional assays may help to uncover the neurobiological basis of memory impairments observed in ASD.

Recently, the BTBR T+tf/J (BTBR) inbred mouse strain has been increasingly used as a rodent model of autism [4, 42, 43]. Extensive tests conducted in multiple independent laboratories have confirmed that BTBR animals display prominent deficits in various social interaction and communication assays, and significantly more repeated and stereotyped behaviours [4, 42, 43]. Thus, the BTBR strain has been considered as a consistent animal model of ASD and affords an opportunity to identify structural and functional changes underlying its robust behavioural phenotype. Notably, in addition to showing behavioural impairments in the core domains of ASD, BTBR mice also display learning and memory deficits in various settings [44–48]. Moreover, using magnetic resonance imaging, it was discovered that the hippocampus had greater relative volume [49, 50] and increased local connectivity [51] in the BTBR animals than the C57BL/6J (B6) strain.

In the present study, we investigated the development of dendritic arbor and neuronal density of pyramidal neurons in hippocampal CA1 region in the BTBR mice. We compared the results to those from the B6 mice, a strain with relatively normal social phenotype and low repetitive behaviours. B6 animals have been routinely used as the control for the BTBR mice in autism-related studies [4, 42, 43], including those addressing learning and memory function [44–48]. Here, we report that during early postnatal development, the BTBR animals had both longer and more complex dendritic arbors, but similar neuronal densities, than the B6 mice. In addition, the observed overgrowth of dendrites in the BTBR animals was associated with a hyper-activation of the ERK signaling pathway in the hippocampus.

## Materials and methods

### Animals

Breeder B6 and BTBR animals were obtained from the Jackson Laboratory (ME) and the lines were maintained at the mouse facility of the Cumming School of Medicine, University of Calgary. Mice were housed in a humidity- and temperature-controlled room with a 12-h light/dark cycle and were fed ad libitum. Neonatal B6 and BTBR male animals at postnatal day 8 (P8) were used. All procedures in this study were performed in accordance with the recommendations in the Canadian Council for Animal Care. The protocol of this study was approved by the Health Sciences Animal Care Committee of the University of Calgary.

### Golgi staining and image acquisition

After euthanization of the animals with overdose of isoflurane, brains were dissected. A Rapid GolgiStain Kit (FD Neurotechnologies, MD) was then used following the manufacturer’s directions. Coronal sections were cut with a cryostat at the thickness of 100 μm. Images of isolated and intact pyramidal neurons in the anterior to middle hippocampal CA1 region were acquired with a Zeiss LSM 510 confocal microscope (ON) using a 20x lens and the bright-field setting. A stack of images with an interval of 1 μm were taken for each cell to include all visible dendritic branches. The stack was then collapsed using the method of minimum intensity projection.

### Dendritic arbor tracing and measurement

Images were imported to the Imaris software (Bitplane, MA) and the dendritic arbors were reconstructed using the Filament function. The total length of the tracing was calculated by Imaris. Parameters of each dendritic arbor, including terminal points, dendritic segments, branch points, and branch levels were also analyzed. ImageJ (NIH, MD) was used to outline and measure the cross-sectional area of the soma.

### Sholl analysis

Dendritic branching pattern was further analyzed by using Sholl analysis in ImageJ [52]. This method creates a series of concentric circles centered at the soma of the neuron in interest, and counts how many times the dendritic arbor intersects the sampling circles, as a function of the distance from the soma. The diameters of the concentric circles were set to have an increment of 20 μm.

### Fluorescent Nissl and immunohistochemical staining and image acquisition

Brain tissue was processed according to previously described methods [53]. Briefly, brains were removed and post-fixed, embedded in gelatin, cryoprotected in 30% sucrose, sectioned in the coronal plane using a cryostat. For Nissl staining, sections including the anterior to middle hippocampus were stained with the NeuroTrace Fluorescent Nissl Stains Kit (Molecular Probes) according to the manufacturer’s protocol. For immunohistochemical staining, same procedures of tissue processing were used, and sections were then incubated with primary (anti-Ctip2, Abcam, MA, 1:500) and secondary antibodies [53]. Images were then acquired with a Zeiss LSM 510 confocal microscope. CA1 pyramidal neurons in 4–6 Nissl-stained sections from each animal were counted at least twice manually and the CA1 layer thickness was measured using the Zen software (Zeiss).

### Western blot

Hippocampus and cortex from brains of P8 mice were dissected and homogenized in RIPA buffer (Pierce Biotechnology, MA) with protease and phosphatase inhibitor cocktails (Roche, QC). Protein concentrations were determined using the BCA protein assay kit (Pierce Biotechnology, MA). 10–50 μg of the protein extract was separated by SDS-PAGE, and transferred to polyvinylidene fluoride (PVDF) membranes. After blocking, blots were incubated with antibodies and visualized with an enhanced chemiluminescence detection system. Bands were imaged and quantified using a ChemiDOC MP gel imaging system (Bio-Rad, CA). The following primary antibodies were used: p-MEK (1:1,000, Cell Signaling, MA, #9154), MEK (1:1,000, Cell Signaling, #9122), p-ERK (1:1,000, Cell Signaling, #9101), ERK (1:1,000, Cell Signaling, #9102), p-CREB (1:2,000, Millipore, MA, #06–519), CREB (1:1,000, Millipore, #06–863), p-4EBP1 (1:200, Cell Signaling, #9459), 4EBP1 (1:200, Cell Signaling, #9452), p-rpS6 (1:1,000, Cell Signaling, #2215 and #2211), rpS6 (1:1,1000, Cell Signaling, #2217), BDNF (1:1,000, Abcam, MA, #ab6201), and actin (1:10,000, Cell Signaling, #4970). After the phosphorylated form of a protein was analyzed, the same membranes were stripped and used to analyze the total form. Finally, the membranes were stripped again and actin was probed. The expression levels of the phosphorylated and total forms of a protein were normalized by actin, and the ratio of the two was computed using the normalized relative expression levels.

### Statistical analysis

Student's *t* test was performed to determine statistical significance between the sets of BTBR and B6 data, assuming two-tailed distribution and two-sample unequal variance. Values represented the mean ± SEM. Two-way repeated measure ANOVA and Holm-Sidak pairwise comparison were used to determine statistically significant differences with the Sholl analysis.

## Results

### CA1 pyramidal neurons had longer dendrites in neonatal BTBR mice

**Fig 1:**
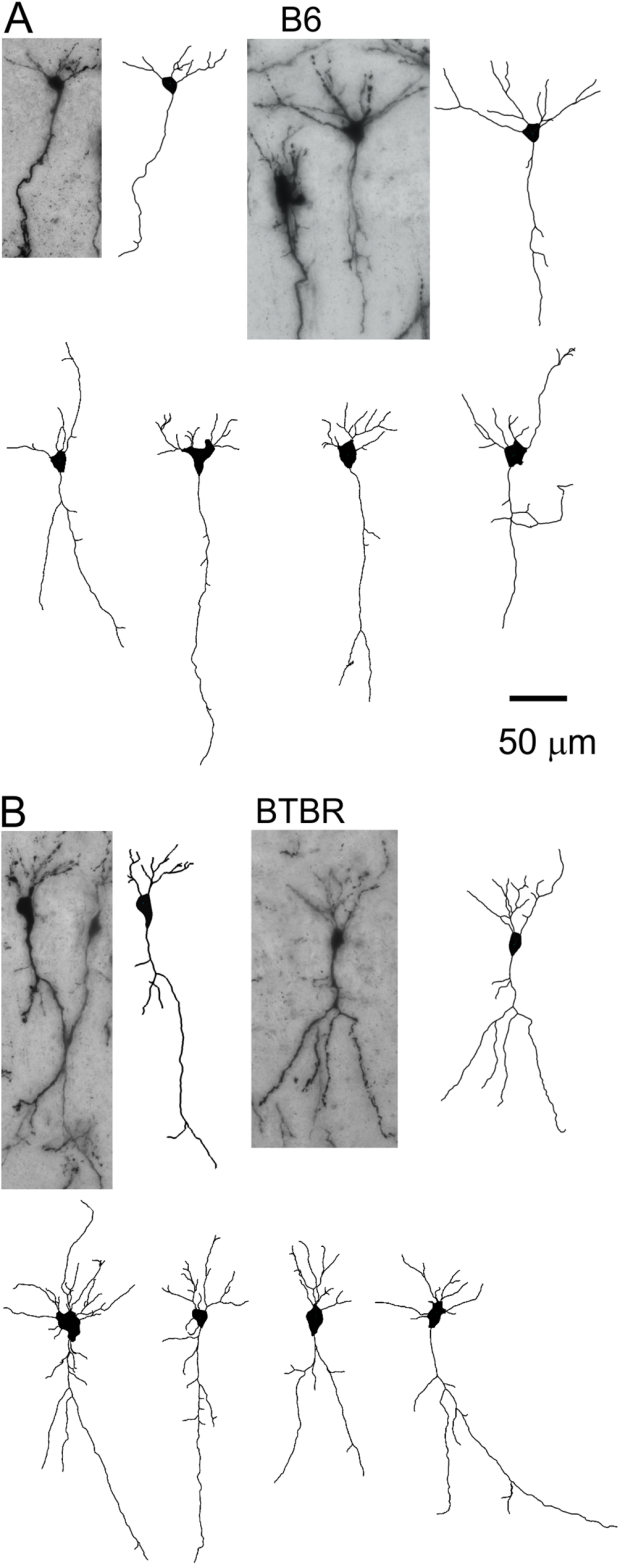
Representative images of Golgi-stained pyramidal neurons in hippocampal CA1 region and the tracings. (**A**) First row: two examples of CA1 pyramidal neurons from the B6 animals, and their corresponding tracings. Second row: more examples of tracings from the B6 mice. (**B**) Similar images of CA1 pyramidal neurons and tracings from the BTBR animals.

**Fig 2:**
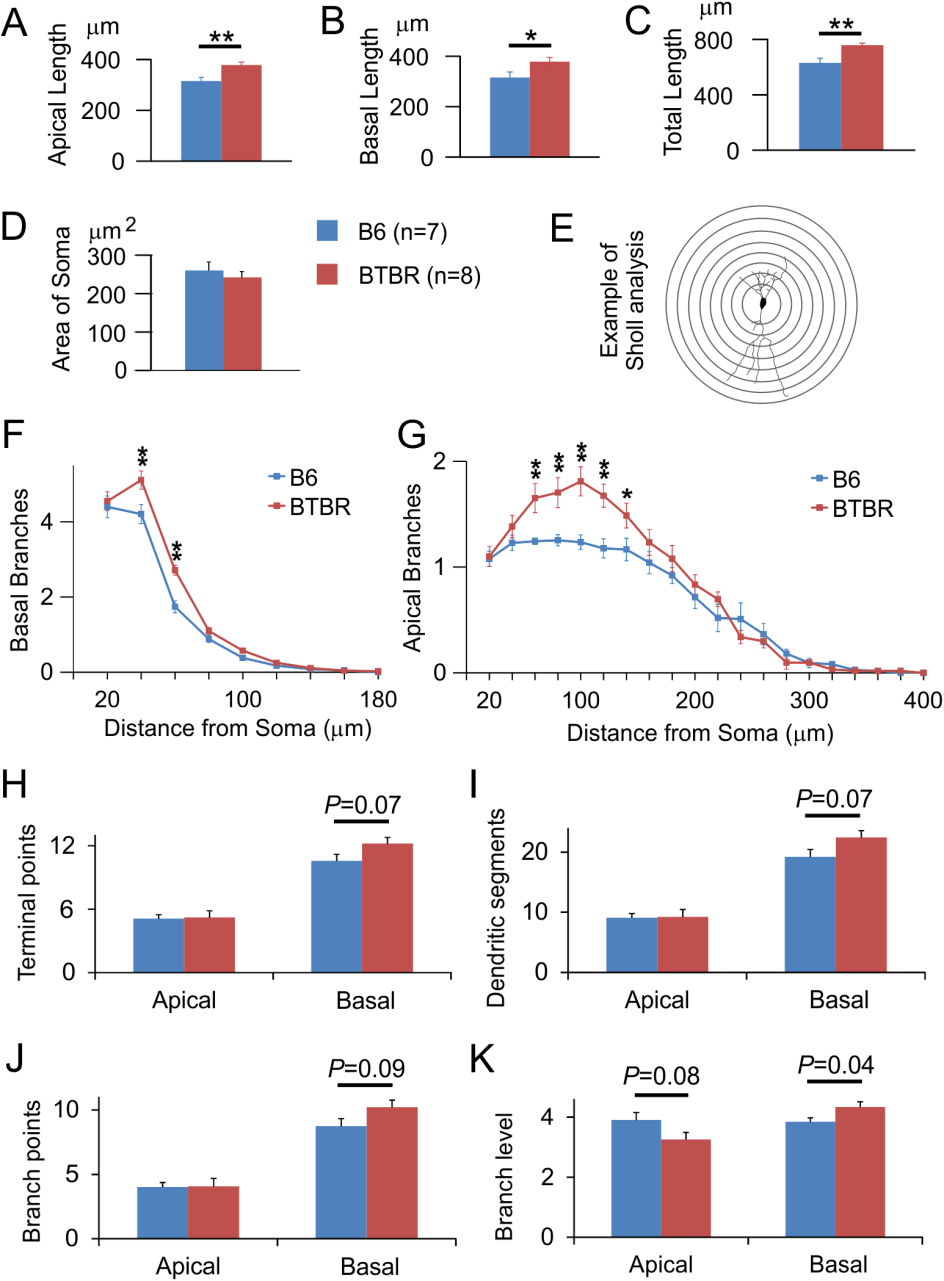
Neonatal BTBR animals had significantly greater dendritic lengths of both apical and basal arbors, and more branching complexity of basal dendrites, compared to the B6 mice. Bar graphs quantifying the length of (**A**) apical dendrites, (**B**) basal dendrites, and (**C**) total dendritic arbor in CA1 pyramidal neurons in B6 versus BTBR animals. Data were collected from 115 cells of seven B6 mice, and 96 cells of eight BTBR mice. Cross-sectional area of the soma was quantified in (**D**). n = 7 animals for B6 and 8 animals for BTBR. *: *P* < 0.05, **: *P* < 0.01 by Student's *t* test. (**E**) Sholl analysis measures the number of intersections between a series of concentric circles centered at the soma and the dendritic arbor of a neuron. Line graphs showing the number of (**F**) basal and (**G**) apical dendritic branches at 20 μm intervals away from the soma in B6 versus BTBR animals. For both basal and apical dendritic tree, *P* < 0.05 comparing B6 vs. BTBR, using two way repeated measure ANOVA. *: *P* < 0.05, **: *P* < 0.001 by Holm-Sidak pairwise comparison. The following parameters of both apical and basal dendritic arbor were further compared between the two strains: (**H**) terminal points, (**I**) dendritic segments, (**J**) branch points, and (**K**) branch level. *P* values were from Student's *t* test.

We first carried out Golgi staining to investigate the dendritic structure in neonatal (P8) mice, which is an age translating to newborn in the human in terms of brain growth [54]. Fig **1** shows representative examples of images taken from Golgi-stained pyramidal neurons and the corresponding tracings in the anterior-to-middle hippocampal CA1 region. In total, 115 pyramidal neurons in seven B6 animals, and 96 neurons in eight BTBR animals, were analysed. Number of analysed neurons in each animal ranged from 8 to 24. We found that the lengths of both apical and basal dendrites, and thus the total length of the dendritic arbor, were significantly increased in the BTBR animals compared with B6 mice (Fig **2A–2C**, *P* = 0.006 for apical dendrites, *P* = 0.041 for basal dendrites, and *P* = 0.008 for total dendritic length). We also analysed the cross-sectional area of the soma of these neurons (n = 4 animals from each strain), but found no significant difference between the two strains (Fig **2D**). Thus, there was specific overgrowth of the dendrites in the BTBR animals but no overall stimulation of cellular growth.

### The structure of the dendritic arbor in BTBR animals was also altered

To analyse the branching pattern of the dendritic arbor in more detail, we next performed Sholl analysis, which counts the intersections of the dendritic tree with a series of concentric circles centred at the soma, as a way to measure the number of branches at a fixed distance along the dendrite (Fig **2E**). We found more branch density at proximal-to-middle range of both apical and basal dendritic arbors in the BTBR mice compared to B6 animals. Specifically, the basal dendrites in the BTBR mice had significantly more branches 40–60 μm away from the soma (Fig **2F**, *P* < 0.001), and the apical dendrites had significantly more branches at 60–140 μm distance from the soma, compared to that seen in the B6 mice (Fig **2G**, **: *P* < 0.01, *: *P* < 0.05). The increases in branch density were consistent with the greater dendritic length in the BTBR mice shown in Fig **2A–2C**, and could be due to either longer branches or more braches, or both, in the BTBR animals. To investigate these possibilities, we further quantified the terminal points, dendritic segments, branch points, and branch level in both apical and basal dendritic arbors. The results revealed that the basal dendrites in BTBR animals showed a significant increase in branch level, and a trend toward an increase in the other parameters, while the apical dendrites showed mostly similar values compared with those in the B6 mice (Fig 2**H–2K**). Together, these data indicate that compared with the B6 animals, the basal dendritic arbor of the BTBR animals was more complex at proximal-to-intermediate distances from the soma, mainly due to oblique dendrites branching more times before terminating. Conversely, the apical dendritic branches of the BTBR animals were longer, but had similar structural complexity as the B6 animals.

### Pyramidal neuron density and thickness of the CA1 neuronal layer were similar between the two strains in both neonatal animals

**Fig 3:**
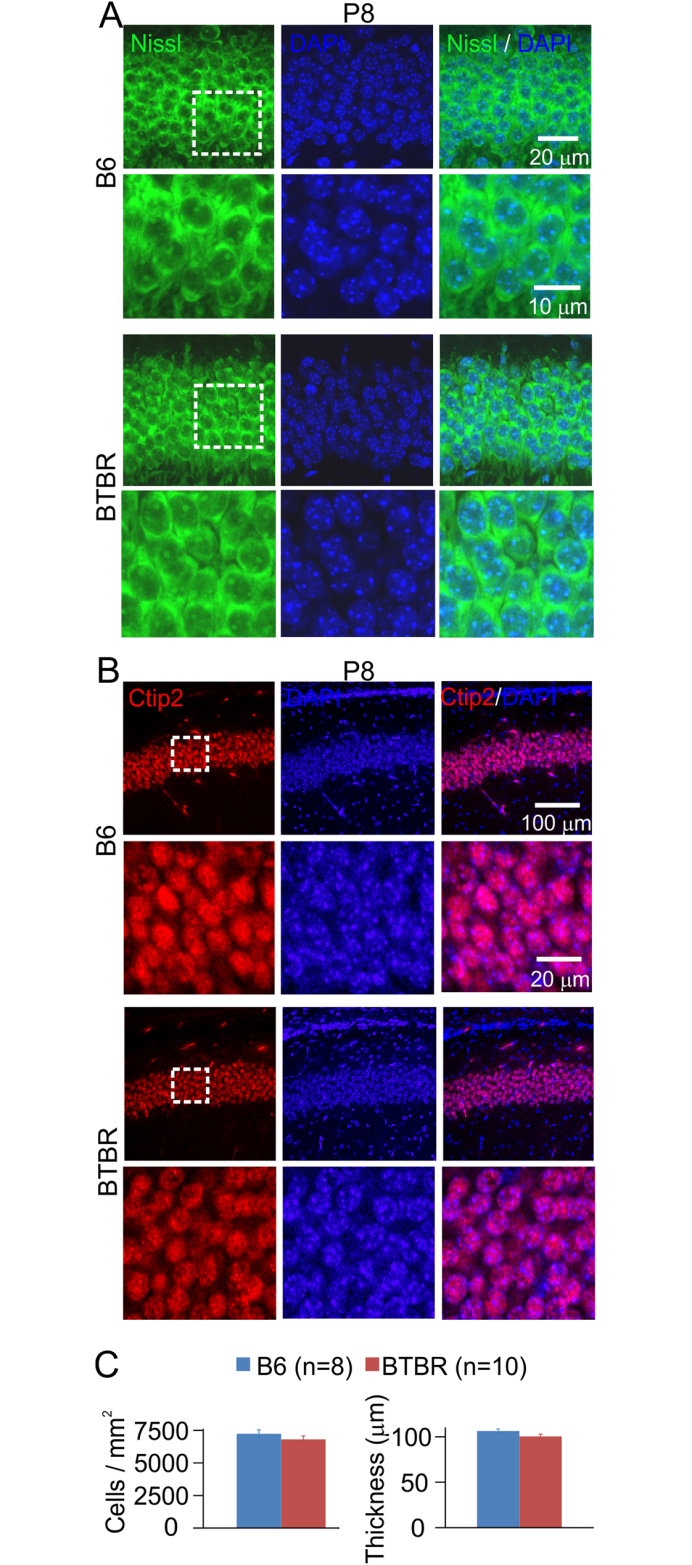
Neonatal B6 and BTBR animals had similar densities of CA1 pyramidal neurons and thickness of the neuronal layer. (**A**) Representative images illustrating fluorescent Nissl (first column) and DAPI (second column) co-staining (third column) of CA1 pyramidal neurons in B6 (upper panels) and BTBR (lower panels) mice. Images in the boxed area are shown in higher magnification below. (**B**) Representative images showing the results of immunohistochemical staining of Ctip2 in CA1 pyramidal neurons. Same organization of panels as in (**A**). (**C**) Bar graphs summarizing the density of pyramidal neurons (left) and the thickness of the CA1 neuronal layer (right) in P8 B6 and BTBR mice measured using Nissl staining. n = 10 animals for each strain.

Dysgenesis of the cerebral cortex has been one of the most consistent abnormalities reported in ASD [1, 55–57], including disrupted layer structure. To examine whether the overgrowth of dendritic arbors in the BTBR animals was associated with overall changes in the cytoarchitecture of the hippocampal CA1 layer, we carried out fluorescent Nissl staining. We did not observe obvious displacement of the pyramidal neurons in the neonatal (P8) BTBR or B6 animals (Fig **3A**). In addition, fluorescent immunohistochemical staining of Ctip2, a zinc finger transcription factor expressed in postmitotic neurons, including hippocampal pyramidal neurons [58, 59], also showed similar pattern between neonatal BTBR and B6 brains (Fig **3B**). Quantification of neuronal density and thickness of the CA1 layer using Nissl-stained sections did not show any significant difference between the two strains (Fig **3C**).

### Activation of the ERK pathway was increased in the BTBR mice

**Fig 4:**
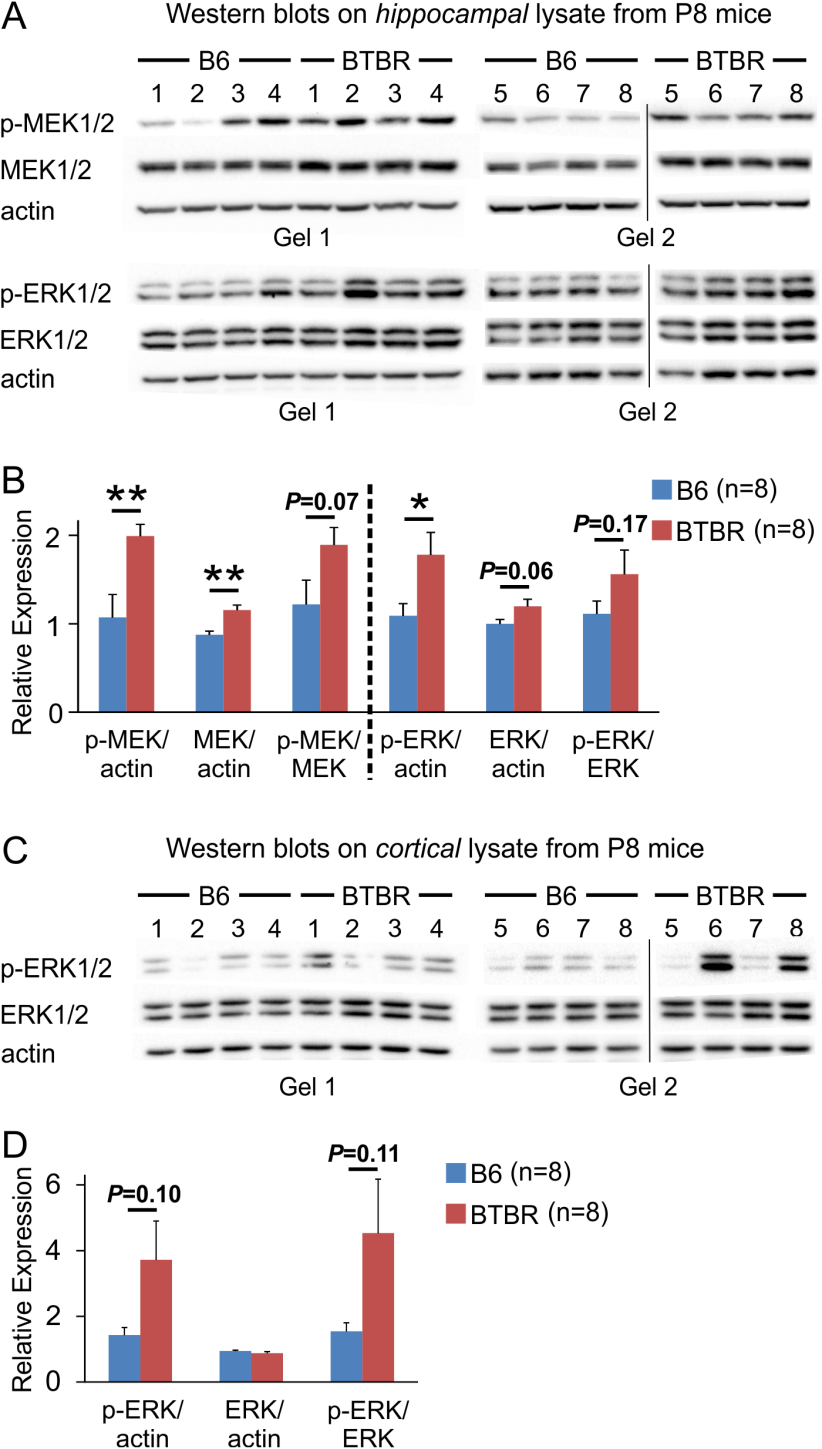
MEK and ERK signaling was up-regulated in the hippocampus of P8 BTBR mice, but not in the cortex. (**A**) Western blots using hippocampal lysates against p-MEK/MEK and p-ERK/ERK. After p-MEK was analyzed, the same membranes were stripped and used to analyze MEK. Finally, the membranes were stripped again and actin was probed. Same approach was used to analyze p-ERK and ERK. (**B**) Quantification of relative expression levels. The expression levels of p-MEK and MEK were normalized by actin, and the ratio of p-MEK to MEK was computed using the normalized relative expression levels of p-MEK and MEK. Same method was used to quantify p-ERK and ERK expression levels and the ratio of the two. (**C**) Western blots using cortical lysates against p-ERK and ERK. Same methods were used as in (**A**). (**D**) Quantification of relative expression levels of p-ERK and ERK in cortical lysates. *: *P* < 0.05, **: *P* < 0.01 by Student's *t* test. Vertical line in the image of (**A**) and (**C**) indicates the position of a lane that was loaded with markers of molecular weight and was cropped out.

To investigate the possible mechanisms that might contribute to the dendritic overgrowth observed in the BTBR animals, we used Western blot to measure the relative expression levels of proteins involved in dendritic growth in P8 animals. We focused on the ERK signaling pathway because it has been shown to regulate dendritic growth and structure, including in mouse models of ASD [22, 25, 32, 33]. In addition, evidence suggests that the ERK cascade may be one of the common, converging signaling pathways dysfunctional in ASD [60–62]. Our results showed that the relative expression levels of both phosphorylated MAPK/ERK kinase (MEK) and ERK were increased in the hippocampal lysate from P8 BTBR animals compared to control B6 animals (Fig **4**). The relative level of total MEK was also increased, while the level of total ERK as well as the ratios of p-MEK to MEK and p-ERK to ERK showed a trend of increase (Fig **4**). As ERK signaling is also critically involved in cortical development [63], we quantified the relative ERK expression levels of cortical homogenates from P8 B6 and BTBR animals. Results showed that although there was a trend of increase in the ratio of p-ERK/actin and p-ERK/ERK expression levels of the BTBR animals compared with B6 mice, the differences did not reach statistical significance, and large variations among the BTBR samples were observed. Thus, the effects of ERK signaling on cortical development in the BTBR mice and how they may influence the development of hippocampal dendritic arbor need to be further explored.

**Fig 5:**
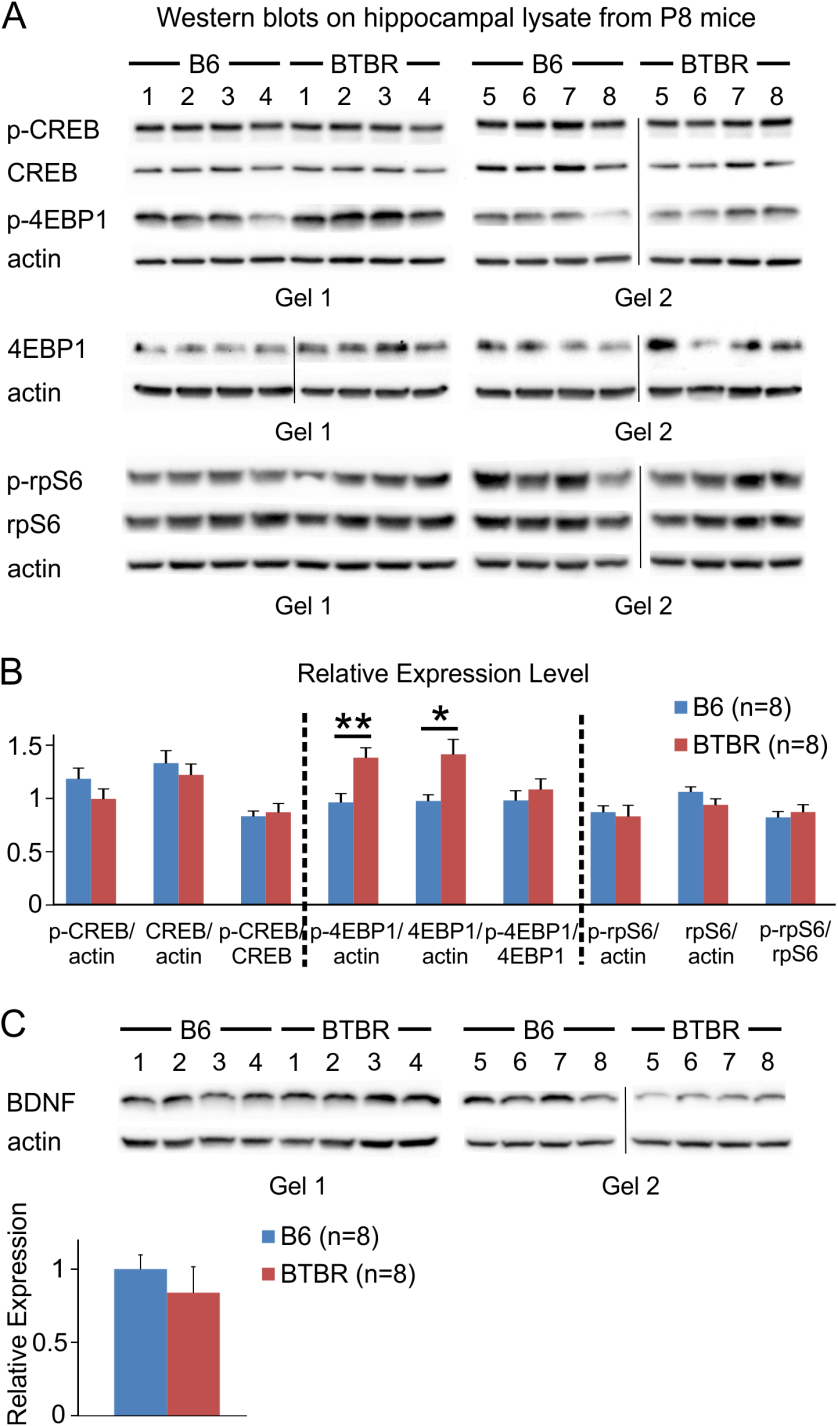
4EBP1 signaling was altered in the hippocampus of P8 BTBR compared to B6 mice, while levels of CREB, rpS6, and BDNF were similar between the two strains. (**A**) Western blots using hippocampal lysates against p-CREB/CREB, p-4EBP1/4EBP1, and p-rpS6/rpS6. Same approach as described in Fig 4 was used, except that 4EBP1 was probed in separate gels from p-4EBP1 because more input protein was required to obtain clear signals (p-4EBP1: 30 μg total protein; 4EBP1: 50 μg total protein). Vertical line in the image indicates the position of a lane that was loaded with markers of molecular weight and was cropped out. (**B**) Quantification of relative expression levels. Same method as described in Fig 4 was used. (**C**) Western blots and relative expression levels of BDNF in the hippocampus of both B6 and BTBR mice. *: *P* < 0.05, **: *P* < 0.01 by Student's *t* test.

It is well known that ERK signaling controls diverse cellular functions through the regulation of both transcriptional and translational processes. Among others, ERK can activate cAMP response element-binding protein (CREB), a transcription factor [64], promote phosphorylation of ribosomal protein S6 (rpS6) and hence translation [65], as well as phosphorylate the eukaryotic translation initiation factor 4E (eIF4E) binding protein 1 (4EBP1), a repressor of translation, and release it from eIF4E [66]. Our data indicated that both phosphorylated and total 4EBP1 was up-regulated in the hippocampus of P8 BTBR mice compared with B6 animals, while the levels of CREB and rpS6 remained unchanged (Fig 5**A** & 5**B**). We also examined the expression level of brain-derived neurotrophic factor (BDNF), which could activate the ERK pathway [67]. However, the hippocampal BDNF level showed a trend toward decrease in the BTBR mice compared to B6 animals (Fig **5C**).

## Discussion

### Potential mechanisms of the observed dendritic overgrowth in the neonatal BTBR animals

In the present study, we observed dendritic overgrowth in the BTBR mouse model of ASD when compared with the B6 mice, while the soma size was similar between the two strains, suggesting that the mechanisms underlying the differences are specific to dendritic development, rather than overall cellular growth. In addition, branching complexity was only increased in the proximal-to-intermediate range of the basal, but not apical, dendritic arbor in the BTBR animals, suggesting differential regulation of specific dendritic compartments [29].

The separation of basal and apical dendrites on all pyramidal neurons suggests that these compartments have distinct functions [68, 69]. For example, basal dendrites of pyramidal neurons are relatively short and close to the soma; thus electrical signals originating there may exert a strong effect on action potential output. Similarly, somatic action potentials could propagate back into the basal dendrites from the axon-soma compartment to influence synaptic plasticity [70]. Furthermore, different dendritic domains receive distinct synaptic inputs and may integrate the inputs differently. In hippocampal CA1 region, the basal and proximal apical dendrites of pyramidal cells receive input primarily from CA3 neurons, whereas the apical tuft receives input from the entorhinal cortex and the thalamus [69]. Studies have also shown that synaptic plasticity could be modulated in a domain-dependent manner. For example, pairing of synaptic and global activity could potentiate synapses on proximal basal dendrites but not on distal ones in neocortical pyramidal neurons. In the distal basal dendrites, synaptic potentiation could occur under unusual conditions when synaptic activation is paired with BDNF application [71]. In addition, it has been shown that synaptic cooperativity at distal dendritic compartments is increased compared with proximal dendritic locations [72]. In this context, it would be interesting to investigate how compartment-specific changes in the dendritic structure observed in the BTBR animals may affect synaptic physiology at different dendritic locations.

Multiple signaling pathways have been implicated in the development of dendritic arbor. For example, several neurotrophic factors have been shown to regulate dendritic growth with distinct effects on various types of neurons. In addition, they appear to exert specific, compartmentalized control over dendritic growth even within the same neuron [27–30]. Neurotrophic factors are thought to regulate one or more downstream intracellular signaling pathways that modulate transcription and/or translation, including the ERK pathway, which is central to many cellular processes. In support of this, ERK has been shown to increase dendritic arborization in an activity-dependent manner [32, 33]. Interestingly, in animal models of ASD, it has recently been reported that ERK promotes increased dendritic complexity in Tsc1^null^ neurons, and reducing ERK signaling rescued dendritic defects [22]. In addition, increased dendritic arborization in cultured cortical neurons has been observed in a mouse model of 16p11.2 duplication, and inhibition of ERK similarly reversed dendritic alterations [25].

Here, we observed increased expression levels of activated MEK and ERK in hippocampal homogenates from P8 BTBR mice. These results are consistent with a previous report showing that Levels of phosphorylated ERK were significantly increased in whole brain lysate of BTBR versus B6 mice at both P0 and P30 [73], and the notion that ERK signaling promotes dendritic arborization described earlier. However, further research is needed to determine the causal relation between ERK hyperactivity and dendritic overgrowth observed here. In addition, both fibroblast growth factor signaling and Wnt signaling have been shown to regulate the development of hippocampus [74–76], and they interact with ERK cascade [59, 63]. Thus, it would be interesting to study these two pathways in the BTBR animals in the future. In an effort to identify the potential targets of the increased ERK signaling, we also quantified the expression levels of phosphorylated as well as total forms of CREB, rpS6, and 4EBP1. We found that protein levels of CREB and rpS6 were similar between BTBR and B6 animals, while both phosphorylated and total 4EBP1 were increased in the BTBR mice. These observations are different from a previous report showing that ERK mediates activity-dependent dendritic growth via CREB signaling [33]. It is possible that ERK may function through multiple pathways to affect dendritic structure, and alternative targets of ERK may need to be identified to elucidate its role in the BTBR model.

Interestingly, previous studies have suggested a link between 4EBP1 and dendritic structure. 4EBP1 has been shown to localize in the dendrites in CA1 region of hippocampus [77], and overexpression of 4EBP1 blocked dendritic overgrowth when the phosphoinositide-3'-kinase-Akt-mammalian target of rapamycin (mTOR) pathway was perturbed [78]. Given that we observed an increase in both phosphorylated and total 4EBP1 protein levels in the BTBR mice, the relation between 4EBP1 signaling and dendritic overgrowth seen here remains to be determined. Since 4EBP1 can be regulated by multiple pathways [79], and given the compartment-specific alterations in dendritic structure as discussed above, it is likely that other mechanisms, such as the mTOR pathway, also play a role in the changes of dendritic structure observed in this study. Notably, dysregulation of eIF4E has been implicated in the pathophysiology of ASD [80–82], supporting a potentially important role of this pathway in the disorder.

Earlier studies have shown that BNDF promotes dendritic development in hippocampal neurons [83, 84]. In the current study, however, we did not observe a significant difference in hippocampal BDNF level between B6 and BTBR animals. Rather, we observed a trend toward decreased BDNF levels in neonatal BTBR mice. The reasons for this difference are uncertain. It is possible that other neurotrophic factors are differentially regulated in the BTBR hippocampus. Consistent with our data, in adult and aged BTBR animals, significant reductions in mRNA or protein levels of BDNF have previously been reported [85–87].

### ERK signaling and ASD

The Ras/Raf/ERK1/2 signaling pathway belongs to the family of mitogen- activated protein kinases (MAPKs) [88]. The MAPK/ERK signaling pathway is highly conserved and mediates the transmission of signals from cell surface receptors to cytoplasmic and nuclear effectors. It plays important roles during multiple stages of brain development [63], and is critically involved in synaptic plasticity, and learning and memory [89, 90]. Notably, 16p11.2 is the most frequent recurrently deleted (0.5%) or duplicated (0.3%) locus in sporadic ASD [91] and this locus includes the ERK1 gene and a gene encoding the major vault protein, which is thought to regulate signaling through the ERK proteins. In addition, network analysis has revealed that autism risk genes converged on cellular cascades related to ERK signaling [61, 62], suggesting that ERK may be a common pathway that is perturbed in the brain of affected individuals [60].

Consistent with genetic studies in patients, and in addition to the reports mentioned above [22, 25, 32, 33], recent laboratory investigations have revealed that genetic manipulation of ERK activity in mice results in abnormal brain development and behaviors associated with ASD, including hippocampus-dependent memory [92]. In addition, in a mouse model of 16p11.2 deletion, ERK activity in cortex and hippocampus was altered, and there was impaired cortical progenitor proliferation and brain cytoarchitecture, as well as memory deficits [93]. Furthermore, transcript knockdown of ASD genes such as Mecp2, Mef2a, Mef2d, *Fmr1*, *Nlgn1*, *Nlgn3*, *Pten*, and *Shank3* has indicated that regulation exerted by a diverse set of ASD-associated genes converges on ERK signaling. In this framework, ERK could act as a hub of multiple pathways involved in neurogenesis, long-term potentiation and synaptic activity [94].

### Structural and functional correlation in the BTBR mouse model of ASD

The BTBR inbred strain is a robust animal model of autism mainly because it displays the core behavioural features that define autism, specifically defective social and communicative behaviours, as well as stereotyped and restricted behaviours and interests [42–44, 95]. In this model, investigators have reported numerous structural and functional aberrations, including changes in genetic and epigenetic expression and regulation, neurotransmission, neuroanatomical and functional connectivity, as well as the immune system. While many of these abnormalities are similar to those found in individuals with autism [4, 42, 43, 96], the causal relations between these changes and the autism-like behaviours largely remain unclear.

Here, we found an overgrowth of the dendritic arbor but similar neuronal density of CA1 pyramidal neurons in neonatal BTBR animals compared with B6 mice. Given the imaging findings that adult BTBR mice have a greater hippocampal volume [49, 50] and increased local connectivity of hippocampus [51] relative to B6 animals, yet overall immunoreactivity of microtubule associated protein MAP2, a marker for dendritic cytoarchitecture, is similar in adult BTBR compared with B6 animals [86], it will be interesting to examine whether the specific, compartmentalized overgrowth of individual neurons in BTBR pups persists into adulthood. Interestingly, a prior study showed that CA1 area of hippocampal slices from the BTBR mice displayed normal long-term potentiation (LTP), paired-pulse facilitation and basal synaptic transmission, as compared to B6 mice. However, hippocampal slices from BTBR mice showed an increased susceptibility to de-potentiation, an activity-induced reversal of LTP [45]. Behaviourally, BTBR mice have also been shown to display learning and memory deficits in various settings [44–48], including reversal learning [44, 46]. It has been established that dendritic structure could influence the firing pattern of neurons [97], and is a major defining factor for induction of synaptic plasticity [14, 15]. Thus, it would be interesting to test whether dendritic overgrowth observed here is linked to altered firing properties of the pyramidal neurons, and whether it contributes to synaptic changes and dysfunctional learning and memory in the BTBR animals.

Although we observed that neuronal density and thickness of the CA1 neuronal layer in a confocal plane were similar between neonatal BTBR and B6 animals, the total volume of the hippocampus and thus the total number of CA1 pyramidal neurons may be different. It will be interesting to use unbiased stereological method to estimate the total number of pyramidal neurons to detect any changes, especially considering that adult BTBR animals have reduced hippocampal commissure [86, 98], but greater relative volume of hippocampus [49, 50].

ASD likely involves widely distributed neural systems and networks. Accordingly, it has been proposed that ASD may represent a disorder of disrupted connectivity [1, 13]. Both over-connectivity and under-connectivity have been reported in ASD patients, and it has been hypothesized that increased local connection and decreased long-range connection may be cardinal features of the disorder and underlie the behavioural impairments [99]. In the BTBR animals, results have shown agenesis of corpus callosum and reduced hippocampal commissure [4, 42, 43], as well as impaired synaptic segregation of retinal ganglion cell input at the lateral geniculate nucleus [100]. Thus, our results support overall altered structural connectivity in both short and long range in this mouse model of ASD, and that these changes in connectivity may underlie its behavioural phenotype.

### Studying autism from a developmental perspective

Changes during early development may contribute significantly to the pathogenesis of ASD, but may not be evident later in life. For example, both functional hypo- and hyper-connectivity of brain networks in ASD have been reported, and by placing these findings in a developmental perspective, a recent theory proposes that hyper-connectivity may be more characteristic of young children with autism, while hypo-connectivity may be more prevalent in adolescents and adults with the disorder [12]. In a related vein, many studies have shown that the size of the corpus callosum is reduced in children and adults with ASD [101]; however, in contrast to these findings, a recent investigation of infants less than 2 years of age showed that the corpus callosum was larger in those who later went on to develop ASD [102]. Together, these observations strongly suggest that changes occurring in the brains of people with autism are dynamic but not uniform across the lifespan, and that early neurodevelopment deserves more attention. In this context, it will be interesting to investigate whether the observed dendritic overgrowth and elevated ERK signaling during neonatal development in the BTBR mouse model contribute to its autism-like behaviour at juvenile and adult stages.

## Conclusions

In this study, we showed that in CA1 pyramidal neurons, dendritic lengths of both apical and basal arbors were greater, and the branching complexity of the basal dendrites was increased, in neonatal BTBR mice compared to B6 animals. No significant differences were found in the density of CA1 pyramidal neurons or the thickness of the neuronal layer between the two strains. We further demonstrated up-regulation of ERK signaling in the hippocampus of neonatal BTBR mice, suggesting that this important intracellular pathway might be involved in the observed dendritic overgrowth. Considering that dendritic pattern is critical for information integration, our data suggest that altered development of dendritic structure could potentially contribute to disrupted connectivity, as well as impaired hippocampal function and behavior observed in this mouse model of autism.

## Abbreviations

BTBR: BTBR T+tf/J strain
B6: C57BL/6J strain
P8: postnatal day 8
ERK: extracellular signal-regulated kinases
MAPK: mitogen-activated protein kinases
CREB: cAMP: response element-binding protein
rpS6: ribosomal protein S6
eIF4E: eukaryotic translation initiation factor 4E
4EBP1: eukaryotic translation initiation factor 4E binding protein 1
BDNF: brain-derived neurotrophic factor
mTOR: mammalian target of rapamycin

## References

[1] DiCicco-Bloom E, Lord C, Zwaigenbaum L, Courchesne E, Dager SR, Schmitz C, et al The developmental neurobiology of autism spectrum disorder. The Journal of neuroscience: the official journal of the Society for Neuroscience. 2006 6 28;26(26):6897–906. doi: 10.1523/JNEUROSCI.1712-06.2006 16807320 10.1523/JNEUROSCI.1712-06.2006 PMC6673916

[2] Geschwind DH. Advances in autism. Annual review of medicine. 2009;60:367–80. Pubmed Central PMCID: 3645857. doi: 10.1146/annurev.med.60.053107.121225 19630577 10.1146/annurev.med.60.053107.121225 PMC3645857

[3] Lai MC, Lombardo MV, Baron-Cohen S. Autism. Lancet. 2014 3 8;383(9920):896–910. doi: 10.1016/S0140-6736(13)61539-1 24074734 10.1016/S0140-6736(13)61539-1

[4] Llaneza DC, DeLuke SV, Batista M, Crawley JN, Christodulu KV, Frye CA. Communication, interventions, and scientific advances in autism: a commentary. Physiology & behavior. 2010 6 1;100(3):268–76. Pubmed Central PMCID: 2860058. doi: 10.1016/j.physbeh.2010.01.003 20093134 10.1016/j.physbeh.2010.01.003 PMC2860058

[5] Aylward EH, Minshew NJ, Field K, Sparks BF, Singh N. Effects of age on brain volume and head circumference in autism. Neurology. 2002 7 23;59(2):175–83. . 12136053 10.1212/wnl.59.2.175

[6] Courchesne E, Karns CM, Davis HR, Ziccardi R, Carper RA, Tigue ZD, et al Unusual brain growth patterns in early life in patients with autistic disorder: an MRI study. Neurology. 2001 7 24;57(2):245–54. . 11468308 10.1212/wnl.57.2.245

[7] Sparks BF, Friedman SD, Shaw DW, Aylward EH, Echelard D, Artru AA, et al Brain structural abnormalities in young children with autism spectrum disorder. Neurology. 2002 7 23;59(2):184–92. . 12136055 10.1212/wnl.59.2.184

[8] Hazlett HC, Poe M, Gerig G, Smith RG, Provenzale J, Ross A, et al Magnetic resonance imaging and head circumference study of brain size in autism: birth through age 2 years. Archives of general psychiatry. 2005 12;62(12):1366–76. doi: 10.1001/archpsyc.62.12.1366 16330725 10.1001/archpsyc.62.12.1366

[9] Schumann CM, Bloss CS, Barnes CC, Wideman GM, Carper RA, Akshoomoff N, et al Longitudinal magnetic resonance imaging study of cortical development through early childhood in autism. The Journal of neuroscience: the official journal of the Society for Neuroscience. 2010 3 24;30(12):4419–27. Pubmed Central PMCID: 2859218. doi: 10.1523/JNEUROSCI.5714-09.2010 20335478 10.1523/JNEUROSCI.5714-09.2010 PMC2859218

[10] Courchesne E, Mouton PR, Calhoun ME, Semendeferi K, Ahrens-Barbeau C, Hallet MJ, et al Neuron number and size in prefrontal cortex of children with autism. Jama. 2011 11 9;306(18):2001–10. doi: 10.1001/jama.2011.1638 22068992 10.1001/jama.2011.1638

[11] Redcay E, Courchesne E. When is the brain enlarged in autism? A meta-analysis of all brain size reports. Biological psychiatry. 2005 7 1;58(1):1–9. doi: 10.1016/j.biopsych.2005.03.026 15935993 10.1016/j.biopsych.2005.03.026

[12] Uddin LQ, Supekar K, Menon V. Reconceptualizing functional brain connectivity in autism from a developmental perspective. Frontiers in human neuroscience. 2013;7:458 Pubmed Central PMCID: 3735986. doi: 10.3389/fnhum.2013.00458 23966925 10.3389/fnhum.2013.00458 PMC3735986

[13] Maximo JO, Cadena EJ, Kana RK. The implications of brain connectivity in the neuropsychology of autism. Neuropsychology review. 2014 3;24(1):16–31. Pubmed Central PMCID: 4059500. doi: 10.1007/s11065-014-9250-0 24496901 10.1007/s11065-014-9250-0 PMC4059500

[14] Hausser M, Spruston N, Stuart GJ. Diversity and dynamics of dendritic signaling. Science. 2000 10 27;290(5492):739–44. . 11052929 10.1126/science.290.5492.739

[15] Jan YN, Jan LY. Branching out: mechanisms of dendritic arborization. Nature reviews Neuroscience. 2010 5;11(5):316–28. Pubmed Central PMCID: 3079328. doi: 10.1038/nrn2836 20404840 10.1038/nrn2836 PMC3079328

[16] Penzes P, Cahill ME, Jones KA, VanLeeuwen JE, Woolfrey KM. Dendritic spine pathology in neuropsychiatric disorders. Nature neuroscience. 2011 3;14(3):285–93. Pubmed Central PMCID: 3530413. doi: 10.1038/nn.2741 21346746 10.1038/nn.2741 PMC3530413

[17] Raymond GV, Bauman ML, Kemper TL. Hippocampus in autism: a Golgi analysis. Acta neuropathologica. 1996;91(1):117–9. . 8773156 10.1007/s004010050401

[18] Feliciano DM, Lin TV, Hartman NW, Bartley CM, Kubera C, Hsieh L, et al A circuitry and biochemical basis for tuberous sclerosis symptoms: from epilepsy to neurocognitive deficits. International journal of developmental neuroscience: the official journal of the International Society for Developmental Neuroscience. 2013 11;31(7):667–78. Pubmed Central PMCID: 3830611. doi: 10.1016/j.ijdevneu.2013.02.008 23485365 10.1016/j.ijdevneu.2013.02.008 PMC3830611

[19] Kwon CH, Luikart BW, Powell CM, Zhou J, Matheny SA, Zhang W, et al Pten regulates neuronal arborization and social interaction in mice. Neuron. 2006 5 4;50(3):377–88. Pubmed Central PMCID: 3902853. doi: 10.1016/j.neuron.2006.03.023 16675393 10.1016/j.neuron.2006.03.023 PMC3902853

[20] Snow WM, Hartle K, Ivanco TL. Altered morphology of motor cortex neurons in the VPA rat model of autism. Developmental psychobiology. 2008 11;50(7):633–9. . 18985861 10.1002/dev.20337

[21] Srivastava DP, Woolfrey KM, Jones KA, Anderson CT, Smith KR, Russell TA, et al An autism-associated variant of Epac2 reveals a role for Ras/Epac2 signaling in controlling basal dendrite maintenance in mice. PLoS biology. 2012;10(6):e1001350 Pubmed Central PMCID: 3383751. doi: 10.1371/journal.pbio.1001350 22745599 10.1371/journal.pbio.1001350 PMC3383751

[22] Zhang L, Bartley CM, Gong X, Hsieh LS, Lin TV, Feliciano DM, et al MEK-ERK1/2-dependent FLNA overexpression promotes abnormal dendritic patterning in tuberous sclerosis independent of mTOR. Neuron. 2014 10 1;84(1):78–91. Pubmed Central PMCID: 4185153. doi: 10.1016/j.neuron.2014.09.009 25277454 10.1016/j.neuron.2014.09.009 PMC4185153

[23] Cochoy DM, Kolevzon A, Kajiwara Y, Schoen M, Pascual-Lucas M, Lurie S, et al Phenotypic and functional analysis of SHANK3 stop mutations identified in individuals with ASD and/or ID. Molecular autism. 2015;6:23 Pubmed Central PMCID: 4455919. doi: 10.1186/s13229-015-0020-5 26045941 10.1186/s13229-015-0020-5 PMC4455919

[24] Crowell B, Lee GH, Nikolaeva I, Dal Pozzo V, D'Arcangelo G. Complex Neurological Phenotype in Mutant Mice Lacking Tsc2 in Excitatory Neurons of the Developing Forebrain(123). eNeuro. 2015 Nov-Dec;2(6). Pubmed Central PMCID: 4676199. doi: 10.1523/ENEURO.0046-15.2015 26693177 10.1523/ENEURO.0046-15.2015 PMC4676199

[25] Blizinsky KD, Diaz-Castro B, Forrest MP, Schurmann B, Bach AP, Martin-de-Saavedra MD, et al Reversal of dendritic phenotypes in 16p11.2 microduplication mouse model neurons by pharmacological targeting of a network hub. Proceedings of the National Academy of Sciences of the United States of America. 2016 7 26;113(30):8520–5. Pubmed Central PMCID: 4968749. doi: 10.1073/pnas.1607014113 27402753 10.1073/pnas.1607014113 PMC4968749

[26] Zhang L, Feliciano DM, Huang T, Zhang S, Bordey A. Hypoxia-inducible factor-1a contributes to dendritic overgrowth in tuberous sclerosis. Neuroscience letters. 2016 1 26;612:43–7. Pubmed Central PMCID: 4728030. doi: 10.1016/j.neulet.2015.11.038 26655465 10.1016/j.neulet.2015.11.038 PMC4728030

[27] McAllister AK, Lo DC, Katz LC. Neurotrophins regulate dendritic growth in developing visual cortex. Neuron. 1995 10;15(4):791–803. . 7576629 10.1016/0896-6273(95)90171-x

[28] McAllister AK, Katz LC, Lo DC. Opposing roles for endogenous BDNF and NT-3 in regulating cortical dendritic growth. Neuron. 1997 5;18(5):767–78. . 9182801 10.1016/s0896-6273(00)80316-5

[29] Niblock MM, Brunso-Bechtold JK, Riddle DR. Insulin-like growth factor I stimulates dendritic growth in primary somatosensory cortex. The Journal of neuroscience: the official journal of the Society for Neuroscience. 2000 6 1;20(11):4165–76. . 10818152 10.1523/JNEUROSCI.20-11-04165.2000 PMC6772633

[30] Alonso M, Medina JH, Pozzo-Miller L. ERK1/2 activation is necessary for BDNF to increase dendritic spine density in hippocampal CA1 pyramidal neurons. Learning & memory. 2004 Mar-Apr;11(2):172–8. Pubmed Central PMCID: 379687. doi: 10.1101/lm.67804 15054132 10.1101/lm.67804 PMC379687

[31] Urbanska M, Gozdz A, Swiech LJ, Jaworski J. Mammalian target of rapamycin complex 1 (mTORC1) and 2 (mTORC2) control the dendritic arbor morphology of hippocampal neurons. The Journal of biological chemistry. 2012 8 31;287(36):30240–56. Pubmed Central PMCID: 3436277. doi: 10.1074/jbc.M112.374405 22810227 10.1074/jbc.M112.374405 PMC3436277

[32] Vaillant AR, Zanassi P, Walsh GS, Aumont A, Alonso A, Miller FD. Signaling mechanisms underlying reversible, activity-dependent dendrite formation. Neuron. 2002 6 13;34(6):985–98. . 12086645 10.1016/s0896-6273(02)00717-1

[33] Ha S, Redmond L. ERK mediates activity dependent neuronal complexity via sustained activity and CREB-mediated signaling. Developmental neurobiology. 2008 12;68(14):1565–79. doi: 10.1002/dneu.20682 18837011 10.1002/dneu.20682

[34] Casanova MF, van Kooten IA, Switala AE, van Engeland H, Heinsen H, Steinbusch HW, et al Minicolumnar abnormalities in autism. Acta neuropathologica. 2006 9;112(3):287–303. doi: 10.1007/s00401-006-0085-5 16819561 10.1007/s00401-006-0085-5

[35] Amaral DG, Schumann CM, Nordahl CW. Neuroanatomy of autism. Trends in neurosciences. 2008 3;31(3):137–45. doi: 10.1016/j.tins.2007.12.005 18258309 10.1016/j.tins.2007.12.005

[36] Casanova MF, Buxhoeveden DP, Switala AE, Roy E. Neuronal density and architecture (Gray Level Index) in the brains of autistic patients. Journal of child neurology. 2002 7;17(7):515–21. doi: 10.1177/088307380201700708 12269731 10.1177/088307380201700708

[37] Oblak AL, Rosene DL, Kemper TL, Bauman ML, Blatt GJ. Altered posterior cingulate cortical cyctoarchitecture, but normal density of neurons and interneurons in the posterior cingulate cortex and fusiform gyrus in autism. Autism research: official journal of the International Society for Autism Research. 2011 6;4(3):200–11. Pubmed Central PMCID: 3110607. doi: 10.1002/aur.188 21360830 10.1002/aur.188 PMC3110607

[38] Fang WQ, Chen WW, Jiang L, Liu K, Yung WH, Fu AK, et al Overproduction of upper-layer neurons in the neocortex leads to autism-like features in mice. Cell reports. 2014 12 11;9(5):1635–43. doi: 10.1016/j.celrep.2014.11.003 25466248 10.1016/j.celrep.2014.11.003

[39] Goh S, Peterson BS. Imaging evidence for disturbances in multiple learning and memory systems in persons with autism spectrum disorders. Developmental medicine and child neurology. 2012 3;54(3):208–13. doi: 10.1111/j.1469-8749.2011.04153.x 22269006 10.1111/j.1469-8749.2011.04153.x

[40] Wang JM, Koldewyn K, Hashimoto R, Schneider A, Le L, Tassone F, et al Male carriers of the FMR1 premutation show altered hippocampal-prefrontal function during memory encoding. Frontiers in human neuroscience. 2012;6:297 Pubmed Central PMCID: 3483622. doi: 10.3389/fnhum.2012.00297 23115550 10.3389/fnhum.2012.00297 PMC3483622

[41] Koldewyn K, Hessl D, Adams J, Tassone F, Hagerman PJ, Hagerman RJ, et al Reduced Hippocampal Activation During Recall is Associated with Elevated FMR1 mRNA and Psychiatric Symptoms in Men with the Fragile X Premutation. Brain imaging and behavior. 2008 1 18;2(2):105–16. Pubmed Central PMCID: 2678852. doi: 10.1007/s11682-008-9020-9 19430586 10.1007/s11682-008-9020-9 PMC2678852

[42] Meyza KZ, Defensor EB, Jensen AL, Corley MJ, Pearson BL, Pobbe RL, et al The BTBR T+ tf/J mouse model for autism spectrum disorders-in search of biomarkers. Behavioural brain research. 2013 8 15;251:25–34. Pubmed Central PMCID: 3529977. doi: 10.1016/j.bbr.2012.07.021 22958973 10.1016/j.bbr.2012.07.021 PMC3529977

[43] Ellegood J, Crawley JN. Behavioral and Neuroanatomical Phenotypes in Mouse Models of Autism. Neurotherapeutics: the journal of the American Society for Experimental NeuroTherapeutics. 2015 7;12(3):521–33. Pubmed Central PMCID: 4489953. doi: 10.1007/s13311-015-0360-z 26036957 10.1007/s13311-015-0360-z PMC4489953

[44] Moy SS, Nadler JJ, Young NB, Perez A, Holloway LP, Barbaro RP, et al Mouse behavioral tasks relevant to autism: phenotypes of 10 inbred strains. Behavioural brain research. 2007 1 10;176(1):4–20. Pubmed Central PMCID: 1857288. doi: 10.1016/j.bbr.2006.07.030 16971002 10.1016/j.bbr.2006.07.030 PMC1857288

[45] MacPherson P, McGaffigan R, Wahlsten D, Nguyen PV. Impaired fear memory, altered object memory and modified hippocampal synaptic plasticity in split-brain mice. Brain research. 2008 5 19;1210:179–88. doi: 10.1016/j.brainres.2008.03.008 18417102 10.1016/j.brainres.2008.03.008

[46] Amodeo DA, Jones JH, Sweeney JA, Ragozzino ME. Differences in BTBR T+ tf/J and C57BL/6J mice on probabilistic reversal learning and stereotyped behaviors. Behavioural brain research. 2012 2 1;227(1):64–72. Pubmed Central PMCID: 3273330. doi: 10.1016/j.bbr.2011.10.032 22056750 10.1016/j.bbr.2011.10.032 PMC3273330

[47] Ribeiro AS, Eales BA, Biddle FG. Short-term and long-term memory deficits in handedness learning in mice with absent corpus callosum and reduced hippocampal commissure. Behavioural brain research. 2013 5 15;245:145–51. doi: 10.1016/j.bbr.2013.02.021 23454853 10.1016/j.bbr.2013.02.021

[48] Yang H, Huh SO, Hong JS. Enhancement of Short-Term Memory by Methyl-6-(Phenylethynyl)-Pyridine in the BTBR T+tf/J Mouse Model of Autism Spectrum Disorder. Endocrinology and metabolism. 2015 3 27;30(1):98–104. Pubmed Central PMCID: 4384677. doi: 10.3803/EnM.2015.30.1.98 25559718 10.3803/EnM.2015.30.1.98 PMC4384677

[49] Ellegood J, Babineau BA, Henkelman RM, Lerch JP, Crawley JN. Neuroanatomical analysis of the BTBR mouse model of autism using magnetic resonance imaging and diffusion tensor imaging. NeuroImage. 2013 4 15;70:288–300. Pubmed Central PMCID: 3595420. doi: 10.1016/j.neuroimage.2012.12.029 23275046 10.1016/j.neuroimage.2012.12.029 PMC3595420

[50] Dodero L, Damiano M, Galbusera A, Bifone A, Tsaftsaris SA, Scattoni ML, et al Neuroimaging evidence of major morpho-anatomical and functional abnormalities in the BTBR T+TF/J mouse model of autism. PloS one. 2013;8(10):e76655 Pubmed Central PMCID: 3797833. doi: 10.1371/journal.pone.0076655 24146902 10.1371/journal.pone.0076655 PMC3797833

[51] Sforazzini F, Bertero A, Dodero L, David G, Galbusera A, Scattoni ML, et al Altered functional connectivity networks in acallosal and socially impaired BTBR mice. Brain structure & function. 2016 3;221(2):941–54. doi: 10.1007/s00429-014-0948-9 25445840 10.1007/s00429-014-0948-9

[52] Ferreira TA, Blackman AV, Oyrer J, Jayabal S, Chung AJ, Watt AJ, et al Neuronal morphometry directly from bitmap images. Nature methods. 2014 10;11(10):982–4. doi: 10.1038/nmeth.3125 25264773 10.1038/nmeth.3125 PMC5271921

[53] Cheng N, Bai L, Steuer E, Belluscio L. Olfactory functions scale with circuit restoration in a rapidly reversible Alzheimer's disease model. The Journal of neuroscience: the official journal of the Society for Neuroscience. 2013 7 24;33(30):12208–17. Pubmed Central PMCID: 3721835. doi: 10.1523/JNEUROSCI.0291-13.2013 23884929 10.1523/JNEUROSCI.0291-13.2013 PMC3721835

[54] Workman AD, Charvet CJ, Clancy B, Darlington RB, Finlay BL. Modeling transformations of neurodevelopmental sequences across mammalian species. The Journal of neuroscience: the official journal of the Society for Neuroscience. 2013 4 24;33(17):7368–83. Pubmed Central PMCID: 3928428. doi: 10.1523/JNEUROSCI.5746-12.2013 23616543 10.1523/JNEUROSCI.5746-12.2013 PMC3928428

[55] Casanova MF, El-Baz AS, Kamat SS, Dombroski BA, Khalifa F, Elnakib A, et al Focal cortical dysplasias in autism spectrum disorders. Acta neuropathologica communications. 2013;1:67 Pubmed Central PMCID: 3893372. doi: 10.1186/2051-5960-1-67 24252498 10.1186/2051-5960-1-67 PMC3893372

[56] Stoner R, Chow ML, Boyle MP, Sunkin SM, Mouton PR, Roy S, et al Patches of disorganization in the neocortex of children with autism. The New England journal of medicine. 2014 3 27;370(13):1209–19. Pubmed Central PMCID: 4499461. doi: 10.1056/NEJMoa1307491 24670167 10.1056/NEJMoa1307491 PMC4499461

[57] Wegiel J, Flory M, Kuchna I, Nowicki K, Ma SY, Imaki H, et al Stereological study of the neuronal number and volume of 38 brain subdivisions of subjects diagnosed with autism reveals significant alterations restricted to the striatum, amygdala and cerebellum. Acta neuropathologica communications. 2014;2:141 Pubmed Central PMCID: 4177256. doi: 10.1186/s40478-014-0141-7 25231243 10.1186/s40478-014-0141-7 PMC4177256

[58] Simon R, Brylka H, Schwegler H, Venkataramanappa S, Andratschke J, Wiegreffe C, et al A dual function of Bcl11b/Ctip2 in hippocampal neurogenesis. The EMBO journal. 2012 6 29;31(13):2922–36. Pubmed Central PMCID: 3395096. doi: 10.1038/emboj.2012.142 22588081 10.1038/emboj.2012.142 PMC3395096

[59] Vithayathil J, Pucilowska J, Goodnough LH, Atit RP, Landreth GE. Dentate Gyrus Development Requires ERK Activity to Maintain Progenitor Population and MAPK Pathway Feedback Regulation. The Journal of neuroscience: the official journal of the Society for Neuroscience. 2015 4 29;35(17):6836–48. Pubmed Central PMCID: 4412899. doi: 10.1523/JNEUROSCI.4196-14.2015 25926459 10.1523/JNEUROSCI.4196-14.2015 PMC4412899

[60] Levitt P, Campbell DB. The genetic and neurobiologic compass points toward common signaling dysfunctions in autism spectrum disorders. The Journal of clinical investigation. 2009 4;119(4):747–54. Pubmed Central PMCID: 2662567. doi: 10.1172/JCI37934 19339766 10.1172/JCI37934 PMC2662567

[61] Pinto D, Delaby E, Merico D, Barbosa M, Merikangas A, Klei L, et al Convergence of genes and cellular pathways dysregulated in autism spectrum disorders. American journal of human genetics. 2014 5 1;94(5):677–94. Pubmed Central PMCID: 4067558. doi: 10.1016/j.ajhg.2014.03.018 24768552 10.1016/j.ajhg.2014.03.018 PMC4067558

[62] Wen Y, Alshikho MJ, Herbert MR. Pathway Network Analyses for Autism Reveal Multisystem Involvement, Major Overlaps with Other Diseases and Convergence upon MAPK and Calcium Signaling. PloS one. 2016;11(4):e0153329 Pubmed Central PMCID: 4824422. doi: 10.1371/journal.pone.0153329 27055244 10.1371/journal.pone.0153329 PMC4824422

[63] Samuels IS, Saitta SC, Landreth GE. MAP'ing CNS development and cognition: an ERKsome process. Neuron. 2009 1 29;61(2):160–7. Pubmed Central PMCID: 3663441. doi: 10.1016/j.neuron.2009.01.001 19186160 10.1016/j.neuron.2009.01.001 PMC3663441

[64] Lyons MR, West AE. Mechanisms of specificity in neuronal activity-regulated gene transcription. Progress in neurobiology. 2011 8;94(3):259–95. Pubmed Central PMCID: 3134613. doi: 10.1016/j.pneurobio.2011.05.003 21620929 10.1016/j.pneurobio.2011.05.003 PMC3134613

[65] Roux PP, Shahbazian D, Vu H, Holz MK, Cohen MS, Taunton J, et al RAS/ERK signaling promotes site-specific ribosomal protein S6 phosphorylation via RSK and stimulates cap-dependent translation. The Journal of biological chemistry. 2007 5 11;282(19):14056–64. Pubmed Central PMCID: 3618456. doi: 10.1074/jbc.M700906200 17360704 10.1074/jbc.M700906200 PMC3618456

[66] Herbert TP, Tee AR, Proud CG. The extracellular signal-regulated kinase pathway regulates the phosphorylation of 4E-BP1 at multiple sites. The Journal of biological chemistry. 2002 3 29;277(13):11591–6. doi: 10.1074/jbc.M110367200 11799119 10.1074/jbc.M110367200

[67] Yoshii A, Constantine-Paton M. Postsynaptic BDNF-TrkB signaling in synapse maturation, plasticity, and disease. Developmental neurobiology. 2010 4;70(5):304–22. Pubmed Central PMCID: 2923204. doi: 10.1002/dneu.20765 20186705 10.1002/dneu.20765 PMC2923204

[68] Spruston N. Pyramidal neurons: dendritic structure and synaptic integration. Nature reviews Neuroscience. 2008 3;9(3):206–21. doi: 10.1038/nrn2286 18270515 10.1038/nrn2286

[69] Amaral DG, Lavenex P. The Hippocampus Book. 2007:37–114.

[70] Zhou WL, Yan P, Wuskell JP, Loew LM, Antic SD. Dynamics of action potential backpropagation in basal dendrites of prefrontal cortical pyramidal neurons. The European journal of neuroscience. 2008 2;27(4):923–36. Pubmed Central PMCID: 2715167. doi: 10.1111/j.1460-9568.2008.06075.x 18279369 10.1111/j.1460-9568.2008.06075.x PMC2715167

[71] Gordon U, Polsky A, Schiller J. Plasticity compartments in basal dendrites of neocortical pyramidal neurons. The Journal of neuroscience: the official journal of the Society for Neuroscience. 2006 12 06;26(49):12717–26. doi: 10.1523/JNEUROSCI.3502-06.2006 17151275 10.1523/JNEUROSCI.3502-06.2006 PMC6674852

[72] Weber JP, Andrasfalvy BK, Polito M, Mago A, Ujfalussy BB, Makara JK. Location-dependent synaptic plasticity rules by dendritic spine cooperativity. Nature communications. 2016 4 21;7:11380 Pubmed Central PMCID: 4844677. doi: 10.1038/ncomms11380 27098773 10.1038/ncomms11380 PMC4844677

[73] Faridar A, Jones-Davis D, Rider E, Li J, Gobius I, Morcom L, et al Mapk/Erk activation in an animal model of social deficits shows a possible link to autism. Molecular autism. 2014;5:57 Pubmed Central PMCID: 4396809. doi: 10.1186/2040-2392-5-57 25874073 10.1186/2040-2392-5-57 PMC4396809

[74] Roelink H. Hippocampus formation: an intriguing collaboration. Current biology: CB. 2000 4 06;10(7):R279–81. . 10753739 10.1016/s0960-9822(00)00407-3

[75] Vaccarino FM, Grigorenko EL, Smith KM, Stevens HE. Regulation of cerebral cortical size and neuron number by fibroblast growth factors: implications for autism. Journal of autism and developmental disorders. 2009 3;39(3):511–20. Pubmed Central PMCID: 2847619. doi: 10.1007/s10803-008-0653-8 18850329 10.1007/s10803-008-0653-8 PMC2847619

[76] Turner CA, Watson SJ, Akil H. The fibroblast growth factor family: neuromodulation of affective behavior. Neuron. 2012 10 04;76(1):160–74. Pubmed Central PMCID: 3476848. doi: 10.1016/j.neuron.2012.08.037 23040813 10.1016/j.neuron.2012.08.037 PMC3476848

[77] Tang SJ, Reis G, Kang H, Gingras AC, Sonenberg N, Schuman EM. A rapamycin-sensitive signaling pathway contributes to long-term synaptic plasticity in the hippocampus. Proceedings of the National Academy of Sciences of the United States of America. 2002 1 8;99(1):467–72. Pubmed Central PMCID: 117583. doi: 10.1073/pnas.012605299 11756682 10.1073/pnas.012605299 PMC117583

[78] Jaworski J, Spangler S, Seeburg DP, Hoogenraad CC, Sheng M. Control of dendritic arborization by the phosphoinositide-3'-kinase-Akt-mammalian target of rapamycin pathway. The Journal of neuroscience: the official journal of the Society for Neuroscience. 2005 12 7;25(49):11300–12. doi: 10.1523/JNEUROSCI.2270-05.2005 16339025 10.1523/JNEUROSCI.2270-05.2005 PMC6725892

[79] Raught B, Gingras AC. eIF4E activity is regulated at multiple levels. The international journal of biochemistry & cell biology. 1999 1;31(1):43–57. . 10216943 10.1016/s1357-2725(98)00131-9

[80] Waltes R, Gfesser J, Haslinger D, Schneider-Momm K, Biscaldi M, Voran A, et al Common EIF4E variants modulate risk for autism spectrum disorders in the high-functioning range. Journal of neural transmission. 2014 9;121(9):1107–16. doi: 10.1007/s00702-014-1230-2 24818597 10.1007/s00702-014-1230-2

[81] Gkogkas CG, Khoutorsky A, Ran I, Rampakakis E, Nevarko T, Weatherill DB, et al Autism-related deficits via dysregulated eIF4E-dependent translational control. Nature. 2013 1 17;493(7432):371–7. Pubmed Central PMCID: 4133997. doi: 10.1038/nature11628 23172145 10.1038/nature11628 PMC4133997

[82] Neves-Pereira M, Muller B, Massie D, Williams JH, O'Brien PC, Hughes A, et al Deregulation of EIF4E: a novel mechanism for autism. Journal of medical genetics. 2009 11;46(11):759–65. doi: 10.1136/jmg.2009.066852 19556253 10.1136/jmg.2009.066852

[83] Tolwani RJ, Buckmaster PS, Varma S, Cosgaya JM, Wu Y, Suri C, et al BDNF overexpression increases dendrite complexity in hippocampal dentate gyrus. Neuroscience. 2002;114(3):795–805. . 12220579 10.1016/s0306-4522(02)00301-9

[84] Choi SH, Li Y, Parada LF, Sisodia SS. Regulation of hippocampal progenitor cell survival, proliferation and dendritic development by BDNF. Molecular neurodegeneration. 2009;4:52 Pubmed Central PMCID: 2806355. doi: 10.1186/1750-1326-4-52 20025751 10.1186/1750-1326-4-52 PMC2806355

[85] Scattoni ML, Martire A, Cartocci G, Ferrante A, Ricceri L. Reduced social interaction, behavioural flexibility and BDNF signalling in the BTBR T+ tf/J strain, a mouse model of autism. Behavioural brain research. 2013 8 15;251:35–40. doi: 10.1016/j.bbr.2012.12.028 23270976 10.1016/j.bbr.2012.12.028

[86] Stephenson DT, O'Neill SM, Narayan S, Tiwari A, Arnold E, Samaroo HD, et al Histopathologic characterization of the BTBR mouse model of autistic-like behavior reveals selective changes in neurodevelopmental proteins and adult hippocampal neurogenesis. Molecular autism. 2011;2(1):7 Pubmed Central PMCID: 3135520. doi: 10.1186/2040-2392-2-7 21575186 10.1186/2040-2392-2-7 PMC3135520

[87] Jasien JM, Daimon CM, Wang R, Shapiro BK, Martin B, Maudsley S. The effects of aging on the BTBR mouse model of autism spectrum disorder. Frontiers in aging neuroscience. 2014;6:225 Pubmed Central PMCID: 4150363. doi: 10.3389/fnagi.2014.00225 25225482 10.3389/fnagi.2014.00225 PMC4150363

[88] Rubinfeld H, Seger R. The ERK cascade: a prototype of MAPK signaling. Molecular biotechnology. 2005 10;31(2):151–74. doi: 10.1385/MB:31:2:151 16170216 10.1385/MB:31:2:151

[89] Davis S, Laroche S. Mitogen-activated protein kinase/extracellular regulated kinase signalling and memory stabilization: a review. Genes, brain, and behavior. 2006;5 Suppl 2:61–72. doi: 10.1111/j.1601-183X.2006.00230.x 16681801 10.1111/j.1601-183X.2006.00230.x

[90] Thomas GM, Huganir RL. MAPK cascade signalling and synaptic plasticity. Nature reviews Neuroscience. 2004 3;5(3):173–83. doi: 10.1038/nrn1346 14976517 10.1038/nrn1346

[91] Krumm N, O'Roak BJ, Shendure J, Eichler EE. A de novo convergence of autism genetics and molecular neuroscience. Trends in neurosciences. 2014 2;37(2):95–105. Pubmed Central PMCID: 4077788. doi: 10.1016/j.tins.2013.11.005 24387789 10.1016/j.tins.2013.11.005 PMC4077788

[92] Pucilowska J, Puzerey PA, Karlo JC, Galan RF, Landreth GE. Disrupted ERK signaling during cortical development leads to abnormal progenitor proliferation, neuronal and network excitability and behavior, modeling human neuro-cardio-facial-cutaneous and related syndromes. The Journal of neuroscience: the official journal of the Society for Neuroscience. 2012 6 20;32(25):8663–77. doi: 10.1523/JNEUROSCI.1107-12.2012 22723706 10.1523/JNEUROSCI.1107-12.2012 PMC6620980

[93] Pucilowska J, Vithayathil J, Tavares EJ, Kelly C, Karlo JC, Landreth GE. The 16p11.2 deletion mouse model of autism exhibits altered cortical progenitor proliferation and brain cytoarchitecture linked to the ERK MAPK pathway. The Journal of neuroscience: the official journal of the Society for Neuroscience. 2015 2 18;35(7):3190–200. doi: 10.1523/JNEUROSCI.4864-13.2015 25698753 10.1523/JNEUROSCI.4864-13.2015 PMC6605601

[94] Lanz TA, Guilmette E, Gosink MM, Fischer JE, Fitzgerald LW, Stephenson DT, et al Transcriptomic analysis of genetically defined autism candidate genes reveals common mechanisms of action. Molecular autism. 2013;4(1):45 Pubmed Central PMCID: 4176301. doi: 10.1186/2040-2392-4-45 24238429 10.1186/2040-2392-4-45 PMC4176301

[95] McFarlane HG, Kusek GK, Yang M, Phoenix JL, Bolivar VJ, Crawley JN. Autism-like behavioral phenotypes in BTBR T+tf/J mice. Genes, brain, and behavior. 2008 3;7(2):152–63. doi: 10.1111/j.1601-183X.2007.00330.x 17559418 10.1111/j.1601-183X.2007.00330.x

[96] Shpyleva S, Ivanovsky S, de Conti A, Melnyk S, Tryndyak V, Beland FA, et al Cerebellar oxidative DNA damage and altered DNA methylation in the BTBR T+tf/J mouse model of autism and similarities with human post mortem cerebellum. PloS one. 2014;9(11):e113712 Pubmed Central PMCID: 4244134. doi: 10.1371/journal.pone.0113712 25423485 10.1371/journal.pone.0113712 PMC4244134

[97] Mainen ZF, Sejnowski TJ. Influence of dendritic structure on firing pattern in model neocortical neurons. Nature. 1996 7 25;382(6589):363–6. doi: 10.1038/382363a0 8684467 10.1038/382363a0

[98] Wahlsten D, Metten P, Crabbe JC. Survey of 21 inbred mouse strains in two laboratories reveals that BTBR T/+ tf/tf has severely reduced hippocampal commissure and absent corpus callosum. Brain research. 2003 5 02;971(1):47–54. . 12691836 10.1016/s0006-8993(03)02354-0

[99] Courchesne E, Pierce K. Why the frontal cortex in autism might be talking only to itself: local over-connectivity but long-distance disconnection. Current opinion in neurobiology. 2005 4;15(2):225–30. doi: 10.1016/j.conb.2005.03.001 15831407 10.1016/j.conb.2005.03.001

[100] Cheng N, Khanbabaei M, Murari K, Rho JM. Disruption of visual circuit formation and refinement in a mouse model of autism. Autism research: official journal of the International Society for Autism Research. 2017 2;10(2):212–23. doi: 10.1002/aur.1687 27529416 10.1002/aur.1687 PMC5324550

[101] Frazier TW, Hardan AY. A meta-analysis of the corpus callosum in autism. Biological psychiatry. 2009 11 15;66(10):935–41. Pubmed Central PMCID: 2783565. doi: 10.1016/j.biopsych.2009.07.022 19748080 10.1016/j.biopsych.2009.07.022 PMC2783565

[102] Wolff JJ, Gerig G, Lewis JD, Soda T, Styner MA, Vachet C, et al Altered corpus callosum morphology associated with autism over the first 2 years of life. Brain: a journal of neurology. 2015 7;138(Pt 7):2046–58. Pubmed Central PMCID: 4492413. doi: 10.1093/brain/awv118 25937563 10.1093/brain/awv118 PMC4492413

